# School types in adolescence and subsequent health and well-being in young adulthood: An outcome-wide analysis

**DOI:** 10.1371/journal.pone.0258723

**Published:** 2021-11-10

**Authors:** Ying Chen, Christina Hinton, Tyler J. VanderWeele

**Affiliations:** 1 Human Flourishing Program, Harvard Institute for Quantitative Social Science, Cambridge, MA, United States of America; 2 Department of Epidemiology, Harvard T.H. Chan School of Public Health, Boston, MA, United States of America; 3 Harvard Graduate School of Education, Cambridge, MA, United States of America; Universidade Federal de Sao Paulo, BRAZIL

## Abstract

While past empirical studies have explored associations between types of primary and secondary schools and student academic achievement, outcomes beyond academic performance remain less well-understood. Using longitudinal data from a cohort of children (N = 12,288, mean age = 14.56 years) of nurses, this study examined associations between the types of schools participants attended in adolescence and a wide range of subsequent psychological well-being, social engagement, character strengths, mental health, health behavior and physical health outcomes. Results in this sample suggested little difference between attending private independent schools and public schools across outcomes in young adulthood. There were, however, notable differences in subsequent outcomes comparing homeschooling and public schools, and possibly some evidence comparing religious schools and public schools. Specifically, there was some evidence that attending religious schools versus public schools was associated with a higher likelihood of frequent religious service attendance and becoming registered voters, a lower risk of overweight/obese, fewer lifetime sexual partners, and a higher risk of subsequently being binge drinkers; however, these associations were not robust to correction for multiple testing. Homeschooling compared with public schooling was associated with subsequently more frequent volunteering (ß = 0.33, 95% CI = 0.15, 0.52), greater forgiveness (ß = 0.31, 95% CI = 0.16, 0.46), and more frequent religious service attendance (Risk Ratio [RR] = 1.51, 95% CI: 1.27, 1.80), and possibly also with greater purpose in life, less marijuana use, and fewer lifetime sexual partners, but negatively associated with college degree attainment (RR = 0.77, 95% CI: 0.67, 0.88) and possibly with greater risk of posttraumatic stress disorder. These results may encourage education stakeholders to consider a wider range of outcomes beyond academic performance in decision-making.

## Introduction

Empirical studies evaluating student outcomes across various types of schools can inform decision-making among policy-makers, educators, parents and other education stakeholders [1]. School experiences in primary and secondary schools may be crucial for shaping individuals’ developmental and well-being trajectories in later life [2], and shaping student well-being is arguably one of the important aims of education [3]. It is, therefore, important to understand students’ long-term achievements and well-being across different aspects of life when comparing various types of primary and secondary schools. Such evidence would further empower decision making among policy-makers, educators, parents and other education stakeholders [1].

While there is considerable variation across individual schools, adolescent schooling can largely be divided into 4 types: public schools, private independent schools, private religious schools and home schooling [4]. According to recent reports, among U.S. adolescents in 2016, approximately 87.0% attended public schools, 8.8% attended private schools and 3.6% were homeschooled [5]. Public schools are mainly funded and regulated by local governments to provide free education to every child [6,7]. In contrast, private schools primarily depend on private sources of funding (e.g., tuition, donation), are operated by private organizations that are either religiously or non-religiously affiliated, and have relatively high autonomy in decision-making such as student enrollment and curriculum development [6,7]. Homeschooling involves providing education at home, which is typically led by parents. Homeschooling can follow a predetermined curriculum (i.e., structured homeschooling), or self-directed natural learning without a fixed curriculum (i.e., unstructured homeschooling) [8,9].

These different types of schools often prioritize different educational goals [9]. For example, schools may aim to support students in developing academic knowledge, intrinsic motivation to learn, social skills and networks, civic engagement, a healthy lifestyle, well-being, good character, or a particular religious faith, with different school types emphasizing each of these goals to greater or lesser extents [10,11]. It is arguably helpful for policy-makers, educators, parents and other education stakeholders to understand associations between school types and student outcomes related to this wide range of educational goals [12–14]. However, to date, the empirical evaluation of student outcomes across school types has, perhaps understandably, been based primarily on academic achievement.

Empirical studies on school types and student outcomes have most often used standardized test scores as the primary outcome for evaluation. The findings from such studies are rather mixed overall [12], with some studies suggesting that students attending private independent schools, private religious schools and structured homeschooling had modestly higher standardized test scores on some disciplines as compared to their peers at public schools [13–17], while other studies did not find such evidence [18–20].

Beyond academic achievement, studies examining school types and student outcomes related to other educational goals are sparse. There has been some prior research exploring various school types in relation to civic engagement and family formation outcomes, with some research suggesting that attending private independent schools and private religious schools is linked with greater civic engagement and more positive family outcomes than attending public schools [21–23], whereas some other research suggested little evidence of such differences [17]. In addition, there has been some prior research on homeschooling versus institutional schooling for a number of student outcomes, with homeschooling associated with greater civic engagement [24], less alcohol and drug use [25,26], better sleep [27], equal or better mental health and well-being [28–31] and equal or better social-emotional skills [24,31,32]. While these studies have contributed to the literature, several methodological concerns remain. For instance, most of these studies had small samples, limited covariate control, and used cross-sectional data, making it difficult to assess evidence for causal effects. More research is needed to gain a comprehensive understanding of associations between various school types and a diverse array of student outcomes, with longitudinal data and rigorous methodologies.

To address these gaps in the literature, we performed an outcome-wide longitudinal analysis [33,34] to compare adolescents attending various types of schools in the years that followed across a wide range of outcomes in their young adulthood, with extensive control of potential confounders (e.g., family socioeconomic status, family environment). The outcomes include multiple indicators of subsequent psychological well-being, social engagement, character strengths, mental health, health behavior and physical health outcomes.

## Methods

### Study population

This study used longitudinal data from the Nurses’ Health Study II (NHSII) [35] and the Growing Up Today Study (GUTS) [36]. Established in 1989, the NHSII cohort enrolled 116,430 female registered nurses aged 25 to 42 years from across the U.S. In 1996, NHSII participants with children between the age of 9 to 14 years old were invited to have their children participate in another cohort GUTS. Invitation letters and questionnaires were then mailed to the children whose mother provided consent. Of them, 16,882 children returned the completed questionnaires at study baseline, thereby assenting to participate. Since then, NHSII and GUTS participants have been followed up through mail or web-based questionnaires annually or biennially [35,36].

In this study, school types were assessed in the GUTS 1999 questionnaire wave (N = 12,288, mean age = 14.56 years); thus, this year was considered as the study baseline. Data on outcome variables were taken from the most recent GUTS questionnaire waves, primarily the 2010 questionnaire wave (mean age = 25.10 years); if the outcome was not assessed at the 2010 wave, we used data from the 2013 or 2007 wave; covariates were mostly assessed at or prior to the 1999 wave (S1 Table provided the timeline regarding the measurements of all variables). Among participants of the 1999 questionnaire, 1,025 individuals had missing data on school type, another 6,711 participants had missing data on at least one covariate (most covariates had less than 18% of missing data); depending on the outcome, another 681 to 1,510 participants had missing outcome data or were lost to follow-up. A multiple imputation procedure was used handle missing data on all variables. This yielded an analytic sample of 12,288 participants, with 2,432 of them being siblings (some families had multiple children enrolled). This study was approved by the Institutional Review Board at the Brigham and Women’s Hospital.

### Exposure assessment

#### School types

Participants were asked to report the types of schools that they were attending in response to the question (GUTS 1999): “What type of school do you attend?” The responses were grouped into 4 categories including public schools, private independent schools, private religious schools, and home schooled. Those who reported not in school or attending universities were excluded from all analyses.

### Outcome assessment

A wide array of outcomes in young adulthood were assessed (primarily in 2010). Such outcomes included indicators of psychological well-being (i.e., life satisfaction, positive affect, self-esteem, emotional regulation), social engagement (i.e., marital status, community engagement, religious service attendance, educational attainment), character strengths (i.e., volunteering, sense of mission, forgiveness, civic engagement), mental health (i.e., depression, anxiety, post-traumatic stress disorder [PTSD]), health behaviors (i.e., current smoking, binge drinking, marijuana or other illicit drug use, prescription drug misuse, number of lifetime sexual partners, early sexual initiation, history of sexually transmitted infections [STIs], short sleep duration, preventive healthcare use), and physical health (i.e., overweight/obesity, a number of physical health problems). Details on the measurement of all outcome variables were provided in the S1 Text.

### Covariate assessment

#### Demographic characteristics

Demographic covariates included participant age (in years), sex (male, female), race/ethnicity (non-Hispanic white, others), geographic region (West, Midwest, South, Northeastern), and puberty development (assessed with the tanner stage score) [36,37]. Maternal demographic covariates were also considered including mother’s age (in years), race/ethnicity (non-Hispanic white, others), and marital status (married, others).

#### Family socioeconomic status (SES)

Multiple indicators of family socioeconomic status were adjusted for including maternal subjective SES in the U.S. and in the community (both assessed with validated scales on a 10-point scale) [38], mother’s current employment status (currently employed, unemployed), father’s educational attainment (high school or less, 2-year college, 4-year college, grad school, non-applicable), pretax household income (1: <$50,000, 2: $50,000-$74,999, 3: $75,000-$99,999, 4: ≥$100,000), census-tract college education rate (used as a continuous variable), and census-tract median income (1: <$50,000, 2: $50,000-$74,999, 3: $75,000-$99,999, 4: ≥$100,000).

#### Family environment factors

The following baseline family environment factors were considered including participant family structure (live with both biological parents, live with a stepparent, others), family dinner frequency (never/sometimes, most days, everyday), religious service attendance (never, less than once/week, at least once/week), maternal relationship satisfaction (retrospectively reported by GUTS participants, assessed with a nine-item validated scale measuring parent-child relationship satisfaction) [39], maternal depression (yes, no), and maternal smoking status (never smoker, former smoker, current smoker).

#### Prior health status or health behaviors

To reduce concerns about reverse causation, the following health characteristics at baseline were adjusted for: depressive symptoms (assessed with the Depression Symptoms Scale of the McKnight Risk Factor Survey) [40], overweight/obesity (yes, no), current cigarette smoking (yes, no), frequent binge drinking (yes, no), marijuana or other illicit drug use (yes, no), prescription drug misuse (yes, no), history of STIs (yes, no), history of early sexual initiation (yes, no), and the number of lifetime sexual partners (a continuous score).

### Statistical analyses

All statistical analyses were performed in SAS 9.4 (tests of statistical significance were two-sided). Analysis of variance and Chi-square tests were used to examine baseline participant characteristics across school types.

In primary analyses, generalized estimated equation (GEE) models with independent covariance structure were used to regress each outcome on school types separately, adjusting for clustering by sibling status. All continuous outcomes were standardized (mean = 0, standard deviation = 1), so the effect estimates were reported in terms of standard deviations in the outcome variables. To account for multiple testing, Bonferroni correction was performed. All models controlled for sociodemographic characteristics, family environment factors, and health status and health-related behaviors at baseline. Because multiple imputation provides a more flexible approach than many other methods of handling missing data [41–43], we performed multiple imputation by chained equations to impute missing data on all variables, with 20 imputed datasets created. As a sensitivity analysis, we also reanalyzed the primary sets of models using complete-case analysis.

A number of other sensitivity analyses were performed. First, because public school qualities are often influenced by district- and state-level characteristics, we reanalyzed the primary sets of models 1) stratified by neighborhood SES first, and then 2) restricting to participants from the 10 states with the highest and the 10 states with the lowest public school ranking [44] separately. Second, because some parents might send their children to religious schools for non-religious reasons [45], we compared students attending religious schools versus public schools, stratified by their frequency of religious service attendance at baseline (considering at least once/week of attendance as a proxy indicator for religiousness). Next, because religious faith is a major reason for homeschooling [6], we compared the home-schooled with those attending religious schools across the outcomes. Lastly, we evaluated the extent to which the associations between school types and various outcomes were robust to potential unmeasured confounding [46–48]. For this purpose, we calculated E-values [47], which represent the minimum strength of association that an unmeasured confounder(s) would need to have with both the exposure and the outcome variables on the risk ratio scale to fully explain away the exposure-outcome associations, above and beyond the measured covariates.

## Results

### Participant characteristics

At study baseline participant age range was 11–19 years, with a mean age of 14.56 years (SD = 1.62). The participants were higher percentage female, primarily non-Hispanic White, mostly had a high level of family SES, and were generally healthy (S1 Table). The majority reported attending public schools (80.56%), followed by private religious schools (9.67%), private independent schools (8.12%), and homeschooling (1.66%). Compared to those at public schools, participants who attended private independent or religious schools generally had a higher level of family SES. Further, participants at religious schools or in homeschooling were more likely to attend religious services, live with both biological parents, have family dinners frequently, and have lower rates of smoking, binge drinking, drug use, maternal depression or maternal smoking at baseline. Consistent with findings in other samples [49], homeschoolers in this sample were more common in the South and Midwest, and their mothers were less likely to be currently employed (Table 1).

**Table 1:** Distribution of participant characteristics by school types at study baseline (the Growing Up Today Study 1999 questionnaire wave, N = 11,263).

	School Types ^a^			
Participant Characteristics	Public school (n = 9,073)	Private school (n = 914)	Religious school (n = 1,089)	Home schooled (n = 187)
***Demographic Factors***				
Age, in years, mean (SD) ^b^	14.53 (1.60)	14.36 (1.62)	14.43 (1.60)	14.32 (1.68)
Male, %	42.30	44.32	38.19	36.07
Non-Hispanic White, %	93.65	91.58	93.62	91.55
Geographic region, %				
West	14.30	18.17	15.51	19.97
Midwest	34.91	36.70	41.65	27.73
South	14.96	13.80	9.96	28.55
Northeast	35.83	31.34	32.88	23.75
Puberty development stage, mean (SD) ^b^	3.93 (1.11)	3.92 (1.10)	3.95 (1.14)	3.92 (1.14)
Mother’s age, mean (SD) ^b^	43.57 (3.56)	44.26 (3.69)	43.75 (3.65)	42.92 (3.39)
Mother’s race/ethnicity (Non-Hispanic White), %	96.17	94.40	95.58	96.80
Mother’s marital status (married), %	91.40	92.82	95.06	95.30
***Socioeconomic Status***				
Mother’s subjective SES in the U.S., mean (SD) ^b^	7.10 (1.29)	7.41 (1.33)	7.26 (1.33)	6.86 (1.25)
Mother’s subjective SES in community, mean (SD) ^b^	7.00 (1.55)	7.22 (1.55)	7.01 (1.55)	7.04 (1.71)
Mother currently employed, %	88.58	82.04	85.10	42.30
Father educational attainment, %				
High school or less	17.79	10.23	13.26	14.36
2-year college	17.38	14.15	13.94	20.01
4-year college	29.80	29.69	31.76	35.08
Grad school	30.34	41.54	37.95	26.61
Non-applicable ^c^	4.69	4.39	3.09	3.94
Pretax household income, %				
<$50,000	13.49	8.29	8.63	40.55
$50,000–$74,999	25.02	19.08	19.52	32.67
$75,000–$99,999	22.98	20.40	21.75	16.12
≥$100,000	38.51	52.23	50.10	10.65
Census tract college education rate, mean (SD) ^b^	0.31 (0.16)	0.35 (0.17)	0.33 (0.16)	0.24 (0.13)
Census tract median income, %				
<$50,000	26.84	21.08	20.31	41.09
$50,000–$74,999	47.09	46.05	50.57	50.13
$75,000–$99,999	19.74	23.35	22.04	8.21
≥$100,000	6.34	9.52	7.07	0.57
***Family Environment Factors***				
Family structure, %				
Live with both biological parents	75.35	74.55	79.90	83.10
Live with a stepparent	4.11	2.00	2.99	3.43
Others	20.54	23.45	17.11	13.47
Family dinner frequency, %				
Never/sometimes	18.54	19.50	18.83	10.61
Most days	41.79	42.74	40.60	26.59
Everyday	39.66	37.76	40.56	62.79
Maternal relationship satisfaction, mean (SD) ^b^	37.72 (7.13)	37.76 (7.28)	37.72 (7.23)	36.57 (7.88)
Religious service attendance, %				
Never	18.07	10.64	2.58	5.02
Less than once/week	28.77	24.18	18.22	9.08
At least once/week	53.16	65.18	79.20	85.90
Maternal depression, %	10.44	10.02	9.92	6.27
Maternal smoking, %				
Never smoker	69.16	70.73	70.27	88.58
Former smoker	23.42	22.59	22.84	8.11
Current smoker	7.41	6.68	6.89	3.31
***Prior health status or health behaviors***				
Prior depressive symptoms, mean (SD) ^b^	1.19 (0.58)	1.24 (0.59)	1.20 (0.58)	1.16 (0.62)
Prior overweight or obesity, %	19.99	19.83	18.58	19.68
Prior cigarette smoking, %	11.04	10.36	10.21	4.87
Prior frequent binge drinking, %	8.14	9.66	6.82	3.97
Prior marijuana use, %	12.29	13.27	9.34	5.86
Prior other illicit drug use, %	4.18	3.61	4.70	4.42
Prior prescription drug misuse, %	6.60	7.82	5.68	6.33
Prior history of STIs, %	0.21	0.00	0.00	0.64
Prior history of early sexual initiation, %	6.20	4.37	3.63	6.33
Prior number of lifetime sexual partners, mean (SD) ^b^	0.19 (0.75)	0.16 (0.71)	0.13 (0.60)	0.16 (0.77)

Abbreviations: SES, socioeconomic status; SD, standard deviation; STIs, sexually-transmitted infections.

^a^ ANOVA or chi-square tests were used to examine the mean levels (SD) of the characteristic or proportion of individuals within each school type category with that characteristic.

^b^ Range of the following participant characteristics were age (range: 11–19 years), puberty development stage (range: 1 to 5), mother’s age (range: 34 to 53), subjective SES in the US (range: 1–10), subjective SES in the community (range: 1–10), census tract college education rate (range: 0%–85%), maternal relationship satisfaction (range: 9–45), prior depressive symptoms (range: 0 to 4), prior number of lifetime sexual partners (range: 0 to 6).

^c^ Father’s education was assessed by the participant’s mother’s report of her spouse’s education level. Those who did not consider themselves as currently having a spouse responded “non-applicable”.

### School types and subsequent health and well-being

There was little difference in subsequent outcomes between adolescents attending private independent schools versus public schools across various health and well-being outcomes examined, except for some evidence that private school students subsequently reported slightly higher levels of forgiveness (β = 0.08, 95% CI: 0.02, 0.15), though the association did not pass the P<0.05 threshold after Bonferroni correction for multiple testing (Table 2).

**Table 2:** School types in adolescence and subsequent health and well-being in young adulthood (Growing Up Today Study from 1999 to 2007, 2010 or 2013 questionnaire wave, N = 12,288^a^).

	School Types ^b^											
	Private school vs. Public school				Religious school vs. Public school				Home schooled vs. Public school			
Health and well-being outcomes	RR	β ^c^	95% CI	P-value	RR	β ^c^	95% CI	P-value	RR	β ^c^	95% CI	P-value
**Psychological Well-being**												
Life satisfaction		0.01	-0.07, 0.10	0.76		0.05	-0.03, 0.13	0.18		0.03	-0.14, 0.21	0.70
Positive affect		0.04	-0.04, 0.11	0.35		0.06	-0.02, 0.14	0.15		0.10	-0.09, 0.29	0.29
Self-esteem		0.01	-0.06, 0.09	0.71		-0.02	-0.10, 0.06	0.67		-0.16	-0.32, 0.01	0.06
Emotional processing		0.00	-0.08, 0.08	0.98		0.01	-0.06, 0.08	0.76		0.02	-0.15, 0.20	0.80
Emotional expression		0.02	-0.07, 0.11	0.67		0.03	-0.04, 0.11	0.40		0.01	-0.17, 0.19	0.92
**Social Engagement**												
Being married	0.95		0.86, 1.04	0.26	0.93		0.85, 1.02	0.12	1.03		0.83, 1.27	0.82
Community engagement	0.98		0.91, 1.06	0.58	0.99		0.92, 1.08	0.89	1.11		0.94, 1.32	0.21
Religious service attendance (≥ once per week)	1.10		0.96, 1.26	0.16	**1.16**		**1.01, 1.32**	**0.03**	**1.51**		**1.27, 1.80**	**<0.002** ^**d**^
Educational attainment (≥college)	0.97		0.94, 1.01	0.18	0.99		0.95, 1.02	0.46	**0.77**		**0.67, 0.88**	**<0.002** ^**d**^
**Character Strengths**												
Frequency of volunteering		0.01	-0.06, 0.08	0.74		-0.02	-0.10, 0.05	0.55		**0.33**	**0.15, 0.52**	**<0.002** ^**d**^
Sense of mission		-0.01	-0.08, 0.06	0.81		-0.02	-0.09, 0.06	0.68		**0.18**	**0.02, 0.35**	**0.03**
Forgiveness of others		**0.08**	**0.02, 0.15**	**0.02**		0.07	-0.01, 0.14	0.07		**0.31**	**0.16, 0.46**	**<0.002** ^**d**^
Registered to vote	1.00		0.98, 1.02	0.98	**1.02**		**1.00, 1.05**	**0.04**	0.98		0.92, 1.04	0.48
**Mental Health**												
Depressive symptoms		0.00	-0.08, 0.09	0.97		0.01	-0.07, 0.09	0.84		0.10	-0.08, 0.27	0.27
Depression diagnosis	0.93		0.76, 1.12	0.43	0.95		0.81, 1.13	0.58	1.21		0.87, 1.67	0.26
Anxiety symptoms		-0.01	-0.08, 0.06	0.78		0.04	-0.03, 0.11	0.23		-0.01	-0.18, 0.15	0.87
Anxiety diagnosis	0.95		0.79, 1.15	0.62	0.95		0.77, 1.16	0.59	1.28		0.87, 1.88	0.22
Probable PTSD	0.91		0.68, 1.23	0.56	0.99		0.75, 1.32	0.96	**1.73**		**1.00, 2.99**	**0.05**
**Health Behaviors**												
Current cigarette smoking	1.09		0.97, 1.23	0.14	1.12		0.99, 1.27	0.08	1.03		0.76, 1.40	0.85
Frequent binge drinking	1.06		0.97, 1.16	0.22	**1.15**		**1.04, 1.27**	**0.01**	0.73		0.51, 1.04	0.08
Marijuana use	1.00		0.93, 1.07	0.96	1.02		0.95, 1.09	0.56	**0.75**		**0.60, 0.95**	**0.02**
Any other illicit drug use	1.01		0.90, 1.14	0.82	1.02		0.90, 1.17	0.72	0.77		0.52, 1.15	0.20
Prescription drug misuse	0.99		0.86, 1.12	0.82	0.92		0.80, 1.07	0.28	0.93		0.68, 1.28	0.67
Number of lifetime sexual partners		-0.04	-0.10, 0.03	0.31		**-0.08**	**-0.14, -0.02**	**0.01**		**-0.20**	**-0.35, -0.06**	**0.01**
Early sexual initiation	1.00		0.83, 1.22	0.97	0.91		0.73, 1.13	0.39	0.85		0.56, 1.29	0.45
History of STIs	0.87		0.71, 1.06	0.17	0.97		0.80, 1.18	0.76	0.75		0.39, 1.45	0.39
Short sleep duration	1.04		0.91, 1.19	0.55	1.10		0.96, 1.26	0.16	0.95		0.69, 1.32	0.77
Preventive healthcare use	0.95		0.89, 1.02	0.13	0.98		0.91, 1.04	0.47	0.87		0.74, 1.03	0.10
**Physical Health**												
Overweight/obesity	0.95		0.87, 1.04	0.28	**0.91**		**0.83, 1.00**	**0.04**	0.98		0.79, 1.21	0.84
No. of physical health problems		0.00	-0.07, 0.08	0.89		-0.01	-0.08, 0.06	0.77		-0.04	-0.21, 0.13	0.64

Abbreviations: RR, risk ratio; CI, confidence interval; PTSD, posttraumatic stress disorder; STIs, sexually transmitted infections.

^a^ The full analytic sample was restricted to those who responded to the Growing Up Today Study 1999 questionnaire wave in which the exposure school type was assessed. Multiple imputation was performed to impute missing data on all variables. In the imputed analytic sample, the sample size for each school type was 9,675 for public school, 1,298 for private non-religious school, 1,126 for religious school, and 189 for home schooled.

^b^ A set of generalized estimating equations were used to regress each outcome on school type separately. All models controlled for participants’ age, sex, race/ethnicity, puberty development, geographic region, mother’s age, mother’s race/ethnicity, mother’s marital status, socioeconomic status (including mother’s subjective socioeconomic status, mother’s employment status, father’s educational attainment, household income, census tract college education rate, and census tract median income), participant family environment (including family structure, family dinner frequency, maternal relationship satisfaction, frequency of religious service attendance, maternal depression, and maternal smoking), and participant prior health status or prior health behaviors (prior depressive symptoms, overweight/obesity, smoking, drinking, marijuana use, other drug use, prescription, drug misuse, number of sexual partners, early sexual initiation, and history of sexually transmitted infections).

^c^ All continuous outcomes were standardized (mean = 0, standard deviation = 1), and β was the standardized effect size.

^d^ p<0.05 after Bonferroni correction (the p value cutoff for Bonferroni correction is p = 0.05/30 outcomes = 0.002).

As compared to public schools, there was some evidence that students at religious schools subsequently had a higher likelihood of frequent religious service attendance and becoming registered voters, a lower risk of overweight/obesity and fewer lifetime sexual partners on average (e.g., β_number of sexual partners_ = -0.08, 95% CI: -0.14, -0.02); however, they were more likely to subsequently be frequent binge drinkers (e.g., RR_binge drinking_ = 1.15, 95% CI: 1.04, 1.27), though such associations again did not reach a *p* < .05 threshold after accounting for multiple testing (Table 2).

Compared to those attending public schools, homeschooled students were subsequently 51% more likely to attend religious services frequently (RR = 1.51, 95% CI: 1.27, 1.80), reported greater frequency of volunteering (β = 0.33, 95% CI: 0.15, 0.52), and had substantially higher levels of forgiveness on average (β = 0.31, 95% CI: 0.16, 0.46), but were 23% less likely to attain a college degree (e.g., RR _attain a college degree_ = 0.77, 95% CI: 0.67, 0.88) in young adulthood; all of these associations also passed the p<0.05 threshold even after Bonferroni correction for multiple testing. There was also some evidence that homeschooled students subsequently reported a higher level of sense of mission in life, lower risks of marijuana use and fewer lifetime sexual partners, but possibly had a higher risk of PTSD; these latter associations, however, passed conventional, but not Bonferroni-corrected, p-value thresholds (Table 2).

### Sensitivity analyses for unmeasured confounding

E-values [47] were calculated for assessing robustness of the observed associations to potential unmeasured confounding (Table 3). There was evidence, for example, that the associations of homeschooling with subsequent volunteering, forgiveness, religious service attendance, and educational attainment were at least moderately robust to unmeasured confounding. For instance, to fully explain away the observed association between homeschool and volunteering above and beyond the measured covariates, an unmeasured confounder associated with both homeschooling and greater likelihood of volunteering by 2.04-fold each on the risk ratio scale could suffice, but weaker joint confounder associations could not; and unmeasured confounding risk ratios of 1.54-fold for both volunteering and home-schooling could suffice to shift the confidence interval to include the null value, but weaker joint confounder could not. Similarly strong E-values were observed with homeschooling in relation to lower education attainment, higher forgiveness, and greater religious service attendance. In contrast, for all comparisons of outcomes for public versus private independent schools, and all comparisons of public versus religious schools, the E-values for the confidence interval were at most 1.24, and often considerably smaller, suggesting modest amounts of confounding could suffice to explain away the observed difference. The only moderately robust evidence to potential unmeasured confounding was thus comparing public schools and homeschooling.

**Table 3:** Robustness to unmeasured confounding (E-values*) for the associations between school types and subsequent health and well-being (Growing Up Today Study [GUTS] from 1999 to 2007, 2010 or 2013 questionnaire wave, N = 12,288).

	Private school		Religious school		Homeschooling	
	Effect estimate†	CI limit‡	Effect estimate†	CI limit‡	Effect estimate†	CI limit‡
Life satisfaction	1.11	1.00	1.27	1.00	1.20	1.00
Positive affect	1.23	1.00	1.30	1.00	1.42	1.00
Self-esteem	1.11	1.00	1.16	1.00	1.58	1.00
Emotional processing	1.00	1.00	1.11	1.00	1.16	1.00
Emotional expression	1.16	1.00	1.20	1.00	1.11	1.00
Being married	1.29	1.00	1.36	1.00	1.21	1.00
Community engagement	1.16	1.00	1.11	1.00	1.46	1.00
Religious service attendance (1x/wk)	1.43	1.00	1.59	1.11	2.39	1.86
Educational attainment (≥college)	1.21	1.00	1.11	1.00	1.92	1.53
Frequency of volunteering	1.11	1.00	1.16	1.00	2.04	1.54
Sense of mission	1.11	1.00	1.16	1.00	1.64	1.14
Forgiveness of others	1.36	1.11	1.33	1.00	1.98	1.59
Registered to vote	1.00	1.00	1.16	1.03	1.16	1.00
Depressive symptoms	1.00	1.00	1.11	1.00	1.42	1.00
Depression diagnosis	1.36	1.00	1.29	1.00	1.71	1.00
Anxiety symptoms	1.11	1.00	1.23	1.00	1.11	1.00
Anxiety diagnosis	1.29	1.00	1.29	1.00	1.88	1.00
Probable PTSD	1.43	1.00	1.11	1.00	2.85	1.07
Current cigarette smoking	1.40	1.00	1.49	1.00	1.21	1.00
Frequent binge drinking	1.31	1.00	1.57	1.24	2.08	1.00
Marijuana use	1.00	1.00	1.16	1.00	2.00	1.29
Any other illicit drug use	1.11	1.00	1.16	1.00	1.92	1.00
Prescription drug misuse	1.11	1.00	1.39	1.00	1.36	1.00
Number of lifetime sexual partners	1.23	1.00	1.36	1.16	1.69	1.29
Early sexual initiation	1.00	1.00	1.43	1.00	1.63	1.00
History of STIs	1.56	1.00	1.21	1.00	2.00	1.00
Short sleep duration	1.24	1.00	1.43	1.00	1.29	1.00
Preventive healthcare use	1.29	1.00	1.16	1.00	1.56	1.00
Overweight/obesity	1.29	1.00	1.43	1.08	1.16	1.00
No. of physical health problems	1.00	1.00	1.11	1.00	1.23	1.00

* See VanderWeele and Ding (ref no.^46^) for the formula for calculating E-values.

† The E-values for effect estimates are the minimum strength of association on the risk ratio scale that an unmeasured confounder would need to have with both the exposure and the outcome to fully explain away the observed exposure-outcome association, conditional on the measured covariates. For example, an unmeasured confounder would need to be associated with both homeschooling and religious service attendance by risk ratios of 2.39 each, above and beyond the measured covariates, to fully explain away the observed association between homeschooling and religious service attendance.

‡ The E-values for the limit of the 95% confidence interval (CI) closest to the null denote the minimum strength of association on the risk ratio scale that an unmeasured confounder would need to have with both the exposure and the outcome to shift the confidence interval to include the null value, conditional on the measured covariates. For example, an unmeasured confounder would need to be associated with both homeschooling and religious service attendance by 1.86-fold each, above and beyond the measured covariates, to shift the lower limit of the confidence interval for the observed association between homeschooling and religious service attendance.

### Other sensitivity analyses

First, reanalyzing the primary models using complete-case analyses yielded similar results as the primary analyses (S2 Table). Second, the analyses stratified by neighborhood SES also yielded similar results as the primary analyses. Specifically, there was little difference between private independent schools and public schools across outcomes among those residing in areas with either low (S3A Table) or high (S3B Table) levels of census-tract median income; magnitudes of the effect estimates comparing religious versus public schools across outcomes were also similar to the primary analyses, but the confidence intervals were wider due to the smaller sample size in each stratum (S3A and S3B Table). Next, the analyses restricting to participants from states with the lowest (S4A Table) and the highest public school rankings (S4B Table) again found little difference between private and public schools in those states. Next, the sensitivity analyses stratified by frequency of religious service attendance suggested that the associations of religious schools (versus public schools) with greater likelihood of registered voting status, fewer lifetime sexual partners and lower risk of overweight/obesity, but elevated risks of binge drinking were slightly stronger among those who attended religious services more frequently (S5 Table). Finally, the analyses comparing homeschooling to religious schools provided some suggestive evidence that the homeschooled adolescents may volunteer more frequently and have a lower risk of marijuana use in their young adulthood (S6 Table).

## Discussion

The present study suggests that for the children of nurses who participated in this study, there was little difference between attending private independent schools versus public schools in subsequent health and well-being outcomes in young adulthood. There was also only modest evidence for differences in subsequent outcomes when comparing private religious schools to public schools. In contrast, there was considerably greater evidence that homeschooling versus public schools was positively associated with several outcomes (e.g., volunteering) but negatively associated with others (e.g., educational attainment). Prior empirical studies comparing student outcomes across various types of schools have primarily used short-term standardized test scores as the outcome for evaluation. This study extends the literature by simultaneously examining multiple long-term health and well-being outcomes using longitudinal data. Below we will comment on relations to prior literature on this topic, but also on the particularities of the sample used in this study.

Consistent with some prior studies suggesting little or only modest differences in test scores comparing private and public school students [19], this study did not find substantial differences in longer-term educational attainment (i.e. college degree). While outcomes beyond academic achievement have been less often investigated, congruent with some of the strongest prior evidence [17], this study also suggested little difference in social connectedness between private versus public school attendants. Likewise, consistent with some prior evidence [17], yet contrary to other studies [21,22], this study also found little difference in subsequent civic engagement comparing private versus public school students. It is possible that private and public schools may differ in outcomes that were not examined in this study, such as students’ subjective schooling experiences, opportunities for parental involvement and parental satisfaction [17]. It is also possible that there may be greater variations within, rather than between, these types of schools. For instance, some important factors that contribute to school performance such as teacher quality, teacher experience, and the availability of after-school programs may vary considerably across individual schools [50].

This study found only relatively modest health and well-being associations comparing attending religious schools versus public schools concerning overweight/obese and lifetime sexual partners. Attending religious schools was associated with a slightly higher risk of frequent binge drinking in young adulthood in this sample. This was surprising as prior research has suggested that religious service attendance during childhood and adolescence is associated with subsequently healthier behaviors in general [51]. However, it may be religious service attendance (rather than religious schooling) that is the primary driver of the overall associations with religious upbringing. Our analyses adjusted for, and stratified by service attendance, while this has not often been accounted for in prior studies of religious schooling [14]. It is, therefore, possible that the associations between religious schooling and health in some prior studies may in fact reflect confounding by religious service attendance, which again evidence suggests is related to subsequent health and well-being [51]. However, if service attendance is itself a part of religious schooling (and possibly the only source of service attendance for some students) then it is also possible that control for service attendance is over-adjustment and may in fact be an integral part of the effects of religious schooling. In any case, the present analysis suggests that it may be religious service attendance, however it is experienced, rather than other aspects of religious schooling that have the more substantial associations with outcomes later in life, at least for the outcomes examined here. Religious knowledge and literacy, which may be the primary motivation for religious schooling for some parents, was not assessed in this study.

The largest differences in our study in subsequent outcomes were between homeschooling and public schools. Congruent with prior studies [31], homeschoolers in this sample (versus those at public schools) were more likely to report subsequently greater character strengths and fewer risky health behaviors. However, homeschooled students were less likely to attain a college degree. While educational attainment may differ between structured and unstructured homeschooling [52], this study did not have data on such subtypes and found that, averaging across these subtypes, and overall homeschoolers had a lower likelihood of attaining a college degree in young adulthood. This may in part reflect lower attainment in learning or less interest in attending college, but it may also reflect the status quo that some U.S. universities have restricted admission policies for the homeschooled [53]. Contrary to prior evidence that homeschoolers (versus public school attenders) typically have equal or greater psychosocial and emotional well-being [31], this study suggested that homeschoolers may have a higher risk of probable PTSD in young adulthood. These contrasting results might in part be attributed to the longitudinal design and the covariate control strategies in this study as compared to prior studies; we were examining outcomes in young adulthood, rather than while the children were still at school and associations could potentially differ for outcomes assessed in the short-run versus the long-run.

There have been controversies over regulations concerning homeschooling and also over whether and what types of public-school services should be made accessible to the homeschooled, with many of the discussions centered around academic resources and extracurricular activities [54,55]. With the growth in internet use, homeschooling has becoming increasingly easier and more popular in the United States [5]. The Covid-19 pandemic has also forced some parents into home-schooling and this may itself alter long-term practices. Although the associations in our study warrant further investigation in future studies, the results here provide some suggestive evidence that support for the psychological well-being of homeschoolers may be worthwhile.

This study is subject to certain limitations. First, the participants were mostly non-Hispanic White and were all children of nurses. Findings of this study may not be generalizable to other populations. Specifically, because all of the students were children of relatively well-educated mothers, this group may have been more able than most to ensure high quality schooling for their children regardless of school type and also more likely to change school type if the particular public or private or religious schools in their area were deemed to be inadequate. The comparisons in this paper pertain to the schools attended by students in this particular sample; they are not comparisons across all U.S. schools. The findings may therefore be most relevant for families who are facing decisions and school dynamics relatively similar to this sample, rather than representative of the general U.S. population. Second, while there may be substantial variation within types of schools [50], we were unable to account for characteristics of individual schools due to the lack of data. However, the homogeneous feature of this sample (all participants were the children of nurses) and the sensitivity analyses stratified by multiple sociodemographic characteristics helped reduce such concerns. Third, the various school types can be further divided into subtypes that may be associated with different outcomes in certain cases [13,56], we could not explore such subtypes here due to a lack of data. For example, we could not examine charter schools separately, which are publicly funded schools with relatively high levels of autonomy in curriculum design, budgets and personnel hiring [57,58], though these are more common now than when school type in this study was assessed. Likewise, we could not examine the subcategories of structured and unstructured homeschooling independently [8]. Further, the sample size of homeschoolers was relatively small (n = 187) in this study, which may have limited our statistical power. However, we nevertheless found associations between homeschooling and several outcomes, even with this more limited statistical power; moreover, we found few differences among any of the other school types, even though the sample sizes were larger.

Despite these limitations, this study provides important evidence concerning associations between school types and a wide range of long-term outcomes. To our knowledge this is the first study that has prospectively examined a wide range of long-term health and well-being outcomes across multiple types of adolescent schooling. Further, this study rigorously accounted for a wide array of covariates that helps reduce concerns about potential confounding, selection bias and reverse causation, which are major methodological concerns in prior studies [59].

School choice is certainly shaped by a variety of factors, such as beliefs, values, and logistical considerations, in addition to a desire for academic learning and educational achievement. A broad range of outcomes, considering numerous aspects of a child’s long-term well-being, is therefore arguably relevant for decision-making. The results of this study might thus help inform policy-makers, educators, parents and other education stakeholders in their decisions by consideration of the evidence on this broader range of educational goals and outcomes.

## Supporting information

S1 Table Distribution of participant characteristics in the full analytic sample. (DOCX)

S2 Table Complete-case analysis on school type in adolescence and subsequent health and well-being in young adulthood. (DOCX)

S3 Table **A.** School type in adolescence and subsequent health and well-being in young adulthood, among those residing in low socioeconomic status neighborhoods. **B.** School type in adolescence and subsequent health and well-being in young adulthood, among those residing in high socioeconomic status neighborhoods.
(DOCX)

S4 Table **A.** School type in adolescence and subsequent health and well-being in young adulthood, among those residing in the top 10 states with the lowest public school ranking. **B.** School type in adolescence and subsequent health and well-being in young adulthood, among those residing in the top 10 states with the highest public school ranking.
(DOCX)

S5 Table Attending religious schools versus public schools in adolescence and subsequent health and well-being in young adulthood, stratified by frequency of religious service attendance in adolescence. (DOCX)

S6 Table Homed schooled vs. attending religious schools in adolescence and subsequent health and well-being in young adulthood. (DOCX)

S1 Text (DOCX)
