# Supplementary material for: School types in adolescence and subsequent health and well-being in young adulthood: An outcome-wide analysis
Source: PLoS One. 2021 Nov 10;16(11):e0258723. doi: 10.1371/journal.pone.0258723 (PMC8580227; doi:10.1371/journal.pone.0258723)
Supplement: S1 Table — (DOCX) [file pone.0258723.s001.docx]

**S1 Table. Distribution of participant characteristics in the full analytic sample (The Growing Up Today Study [GUTS] and The Nurses’ Health Study II [NHSII], N=12,288)**

|  | **Questionnaire**  **Wave** | **Mean (SD) or %** |
| --- | --- | --- |
| **Exposure** (GUTS, wave 1999) |  |  |
| School type, % | 1999 |  |
| Public school |  | 80.56 |
| Private independent school |  | 8.12 |
| Private religious school |  | 9.67 |
| Home schooling |  | 1.66 |
|  |  |  |
| **Outcomes** (GUTS, wave 2010 or 2013) |  |  |
| Psychological Well-being |  |  |
| Life satisfaction (range: 1-6) | 2010 | 4.44 (1.02) |
| Positive affect (range: 10- 60) | 2010 | 40.00 (8.77) |
| Self-esteem (range: 10-40) | 2010 | 33.72 (4.98) |
| Emotional processing (range: 4-16) | 2010 | 11.22 (2.76) |
| Emotional expression (range: 4-16) | 2010 | 10.65 (3.01) |
| Social Engagement |  |  |
| Being married, % | 2010 | 37.98 |
| Community engagement, % | 2010 | 46.50 |
| Religious service attendance (≥ once per week), % | 2007 | 20.21 |
| Educational attainment (≥college), % | 2010 | 80.22 |
| Character Strengths |  |  |
| Frequency of volunteering (range: 1-4) | 2007 | 1.83 (0.87) |
| Sense of mission (range: 1-4) | 2007 | 3.06 (0.73) |
| Forgiveness of others (range: 1-4) | 2007 | 2.93 (0.86) |
| Registered to vote, % | 2007 | 89.45 |
| Mental Health |  |  |
| Depressive symptoms (range: 0-30) | 2010 | 7.25 (4.63) |
| Depression diagnosis, % | 2013 | 17.07 |
| Anxiety symptoms (range: 9-54) | 2010 | 22.25 (8.36) |
| Anxiety diagnosis, % | 2013 | 13.81 |
| Probable PTSD, % | 2007 | 7.57 |
| Health Behaviors |  |  |
| Current cigarette smoking, % | 2010 | 24.83 |
| Frequent binge drinking, % | 2010 | 30.88 |
| Marijuana use, % | 2010 | 55.13 |
| Any other illicit drug use, % | 2010 | 22.55 |
| Prescription drug misuse, % | 2010 | 23.43 |
| Number of lifetime sexual partners (range: 0-8) | 2007 | 3.12 (2.58) |
| Early sexual initiation, % | 2007 | 10.89 |
| History of STIs, % | 2013 | 12.68 |
| Short sleep duration, % | 2010 | 21.69 |
| Preventive healthcare use, % | 2010 | 57.52 |
| Physical Health |  |  |
| Overweight/obesity, % | 2010 | 36.97 |
| No. of physical health problems (range: 0-4) | 2010 | 0.36 (0.59) |
| Hypertension, % | 2010 | 5.37 |
| High cholesterol | 2010 | 8.25 |
| Diabetes, % | 2010 | 0.92 |
| Cancer, % | 2010 | 1.05 |
| Asthma, % | 2010 | 20.70 |
|  |  |  |
| **Covariates** (offspring covariates assessed in GUTS, and maternal covariates assessed in NHSII) | | |
| Age (range: 11-19) | 1999 | 14.56 (1.62) |
| Male, % | 1996 | 42.82 |
| Non-Hispanic White, % | 1996 | 93.23 |
| Geographic region, % | 1999 |  |
| West |  | 14.80 |
| Midwest |  | 5.53 |
| South |  | 14.63 |
| Northeast |  | 35.04 |
| Puberty development stage, (range: 1-5) | 1998 | 3.94 (1.11) |
| Mother’s age (range: 34-53) | NHSII 1999 | 43.64 (3.57) |
| Mother’s race/ethnicity (Non-Hispanic White), % | NHSII 1989 | 95.84 |
| Mother’s marital status (married), % | NHSII 1997 | 91.71 |
| Mother’s subjective SES in the U.S. (range: 1-10) | NHSII 2001 | 7.13 (1.30) |
| Mother’s subjective SES in community (range: 1-10) | NHSII 2001 | 7.02 (1.55) |
| Mother currently employed, % | NHSII 1997 | 87.07 |
| Father educational attainment, % | NHSII 1999 |  |
| High school or less |  | 16.64 |
| 2-year college |  | 16.90 |
| 4-year college |  | 30.88 |
| Grad school |  | 31.77 |
| Non-applicable |  | 4.60 |
| Pretax household income, % | NHSII 2001 |  |
| <$50,000 |  | 12.93 |
| $50,000–$74,999 |  | 23.82 |
| $75,000–$99,999 |  | 22.53 |
| ≥$100,000 |  | 40.72 |
| Census tract college education rate (range: 0-0.85) | NHSII 2001 | 0.32 (0.16) |
| Census tract median income, % | NHSII 2001 |  |
| <$50,000 |  | 25.79 |
| $50,000–$74,999 |  | 47.59 |
| $75,000–$99,999 |  | 19.95 |
| ≥$100,000 |  | 6.68 |
| Family structure, % | 1998 |  |
| Live with both biological parents |  | 71.00 |
| Live with a stepparent |  | 3.58 |
| Others |  | 25.42 |
| Family dinner frequency, % | 1997 |  |
| Never/sometimes |  | 18.57 |
| Most days |  | 41.74 |
| Everyday |  | 39.69 |
| Maternal relationship satisfaction (range: 9-45) | 2005 | 37.72 (7.16) |
| Religious service attendance, % | 1999 |  |
| Never |  | 15.61 |
| Less than once/week |  | 26.94 |
| At least once/week |  | 57.46 |
| Maternal depression, % | NHSII 1997 | 10.31 |
| Maternal smoking, % | NHSII 1997 |  |
| Never smoker |  | 69.70 |
| Former smoker |  | 23.13 |
| Current smoker |  | 7.17 |
| Prior depressive symptoms (range: 0-48) | 1999 | 1.20 (0.58) |
| Prior overweight or obesity, % | 1998 | 19.61 |
| Prior cigarette smoking, % | 1997 | 11.18 |
| Prior frequent binge drinking, % | 1998 | 8.28 |
| Prior marijuana use, % | 1999 | 11.95 |
| Prior other illicit drug use, % | 1999 | 4.17 |
| Prior prescription drug misuse, % | 1999 | 6.59 |
| Prior history of STIs, % | 1999 | 0.18 |
| Prior history of early sexual initiation, % | 1999 | 5.79 |
| Prior number of lifetime sexual partners (range: 0-6) | 1999 | 0.18 (0.73) |
