# Supplementary material for: School types in adolescence and subsequent health and well-being in young adulthood: An outcome-wide analysis
Source: PLoS One. 2021 Nov 10;16(11):e0258723. doi: 10.1371/journal.pone.0258723 (PMC8580227; doi:10.1371/journal.pone.0258723)
Supplement: S2 Table — (DOCX) [file pone.0258723.s002.docx]

**S2 Table. Complete-case analysis on school type in adolescence and subsequent health and well-being in young adulthood (Growing Up Today Study from 1999 to 2007, 2010 or 2013 questionnaire wave, N ranged from 4,209 to 5,512^a^)**

|  | **School Types** ^b^ | | | | | | | | | | | | | |
| --- | --- | --- | --- | --- | --- | --- | --- | --- | --- | --- | --- | --- | --- | --- |
|  | Private school vs. public school | | | |  | Religious school vs. public school | | | |  | Home schooled vs. public school | | | |
| Health and well-being outcomes | RR | β ^c^ | 95% CI | P-value |  | RR | β ^c^ | 95% CI | P-value |  | RR | β ^c^ | 95% CI | P-value |
| **Psychological Well-being** |  |  |  |  |  |  |  |  |  |  |  |  |  |  |
| Life satisfaction |  | -0.01 | -0.12, 0.09 | 0.80 |  |  | 0.02 | -0.07, 0.11 | 0.71 |  |  | 0.02 | -0.20, 0.24 | 0.86 |
| Positive affect |  | 0.01 | -0.09, 0.12 | 0.81 |  |  | 0.01 | -0.08, 0.10 | 0.78 |  |  | 0.13 | -0.09, 0.35 | 0.24 |
| Self-esteem |  | 0.03 | -0.07, 0.13 | 0.56 |  |  | -0.04 | -0.13, 0.05 | 0.35 |  |  | -0.19 | -0.40, 0.03 | 0.09 |
| Emotional processing |  | 0.00 | -0.10, 0.11 | 0.93 |  |  | 0.03 | -0.06, 0.12 | 0.51 |  |  | -0.04 | -0.27, 0.19 | 0.74 |
| Emotional expression |  | 0.04 | -0.07, 0.14 | 0.48 |  |  | 0.05 | -0.04, 0.14 | 0.28 |  |  | 0.00 | -0.23, 0.24 | 0.99 |
| **Social Engagement** |  |  |  |  |  |  |  |  |  |  |  |  |  |  |
| Being married | 0.92 |  | 0.81, 1.05 | 0.23 |  | 0.92 |  | 0.82, 1.03 | 0.16 |  | 1.05 |  | 0.82, 1.35 | 0.68 |
| Religious service attendance  (≥ once per week) | 1.03 |  | 0.86, 1.23 | 0.77 |  | 1.12 |  | 0.97, 1.29 | 0.11 |  | **1.53** |  | **1.22, 1.91** | **<.002^d^** |
| Educational attainment (≥college) | 0.97 |  | 0.92, 1.01 | 0.14 |  | 1.02 |  | 0.98, 1.05 | 0.39 |  | **0.72** |  | **0.60, 0.88** | **<.002^d^** |
| **Character Strengths** |  |  |  |  |  |  |  |  |  |  |  |  |  |  |
| Frequency of volunteering |  | 0.01 | -0.08, 0.11 | 0.77 |  |  | -0.07 | -0.16, 0.01 | 0.10 |  |  | **0.29** | **0.05, 0.53** | **0.02** |
| Sense of mission |  | -0.03 | -0.12, 0.06 | 0.49 |  |  | -0.04 | -0.12, 0.05 | 0.40 |  |  | 0.21 | 0.00, 0.43 | 0.05 |
| Forgiveness of others |  | **0.10** | **0.01, 0.20** | **0.04** |  |  | 0.02 | -0.06, 0.10 | 0.61 |  |  | **0.31** | **0.14, 0.48** | **<.002^d^** |
| Registered to vote | 1.00 |  | 0.97, 1.03 | 0.97 |  | 1.02 |  | 0.99, 1.05 | 0.12 |  | 0.95 |  | 0.87, 1.04 | 0.25 |
| **Mental Health** |  |  |  |  |  |  |  |  |  |  |  |  |  |  |
| Depressive symptoms |  | 0.07 | -0.04, 0.18 | 0.23 |  |  | 0.01 | -0.08, 0.11 | 0.77 |  |  | 0.16 | -0.06, 0.37 | 0.16 |
| Depression diagnosis | 0.94 |  | 0.75, 1.19 | 0.62 |  | 0.89 |  | 0.71, 1.12 | 0.32 |  | 1.13 |  | 0.70, 1.84 | 0.61 |
| Anxiety symptoms |  | -0.03 | -0.13, 0.06 | 0.48 |  |  | 0.09 | 0.00, 0.19 | 0.05 |  |  | -0.08 | -0.27, 0.11 | 0.39 |
| Anxiety diagnosis | 0.95 |  | 0.73, 1.24 | 0.72 |  | 0.95 |  | 0.74, 1.23 | 0.72 |  | 1.45 |  | 0.90, 2.35 | 0.13 |
| **Health Behaviors** |  |  |  |  |  |  |  |  |  |  |  |  |  |  |
| Current cigarette smoking | **1.27** |  | **1.09, 1.49** | **0.002** |  | 1.16 |  | 0.99, 1.36 | 0.06 |  | 0.86 |  | 0.52, 1.41 | 0.55 |
| Frequent binge drinking | 1.11 |  | 0.97, 1.27 | 0.14 |  | **1.22** |  | **1.08, 1.38** | **<.002^d^** |  | **0.42** |  | **0.22, 0.78** | **0.01** |
| Marijuana use | 1.02 |  | 0.94, 1.11 | 0.61 |  | 1.06 |  | 0.97, 1.15 | 0.18 |  | **0.68** |  | **0.50, 0.92** | **0.01** |
| Any other illicit drug use | 1.05 |  | 0.88, 1.25 | 0.61 |  | 1.11 |  | 0.93, 1.34 | 0.25 |  | 0.61 |  | 0.34, 1.11 | 0.11 |
| Prescription drug misuse | 1.02 |  | 0.85, 1.21 | 0.84 |  | 0.89 |  | 0.73, 1.08 | 0.23 |  | 0.88 |  | 0.56, 1.37 | 0.56 |
| Number of sexual partners |  | 0.01 | -0.08, 0.10 | 0.75 |  |  | -0.07 | -0.15, 0.01 | 0.07 |  |  | **-0.19** | **-0.39, 0.00** | **0.05** |
| Early sexual initiation | 0.87 |  | 0.64, 1.18 | 0.37 |  | 0.86 |  | 0.65, 1.13 | 0.27 |  | 0.87 |  | 0.49, 1.56 | 0.65 |
| History of STIs | 0.88 |  | 0.66, 1.17 | 0.39 |  | 0.99 |  | 0.76, 1.27 | 0.91 |  | 0.87 |  | 0.41, 1.84 | 0.72 |
| Short sleep duration | 0.98 |  | 0.80, 1.20 | 0.84 |  | 1.08 |  | 0.90, 1.29 | 0.40 |  | 1.01 |  | 0.65, 1.58 | 0.96 |
| Preventive healthcare use | 0.93 |  | 0.85, 1.02 | 0.13 |  | 1.00 |  | 0.93, 1.08 | 0.90 |  | 0.97 |  | 0.78, 1.20 | 0.77 |
| **Physical Health** |  |  |  |  |  |  |  |  |  |  |  |  |  |  |
| Overweight/obesity | 1.00 |  | 0.87, 1.13 | 0.95 |  | 0.89 |  | 0.77, 1.01 | 0.08 |  | 0.92 |  | 0.69, 1.23 | 0.57 |
| No. of physical health problems |  | 0.01 | -0.09, 0.11 | 0.82 |  |  | -0.05 | -0.14, 0.04 | 0.27 |  |  | -0.05 | -0.25, 0.16 | 0.66 |

Abbreviations: RR, risk ratio; CI, confidence interval.

^a^ The full analytic sample was restricted to those who had complete data on the exposure, all covariates and the outcome variable under investigation. The sample size for each analysis varies, depending on the outcome variable.

^b^ A set of generalized estimating equations were used to regress each outcome on school type separately. All models controlled for participants’ age, sex, race/ethnicity, puberty development, geographic region, mother’s age, mother’s race/ethnicity, mother’s marital status, socioeconomic status (including mother’s subjective socioeconomic status, mother’s employment status, father’s educational attainment, household income, census tract college education rate, and census tract median income), participant family environment (including family structure, family dinner frequency, maternal relationship satisfaction, frequency of religious service attendance, maternal depression, and maternal smoking), and participant prior health status or prior health behaviors (prior depressive symptoms, overweight/obesity, smoking, drinking, marijuana use, other drug use, prescription, drug misuse, number of sexual partners, early sexual initiation, and history of sexually transmitted infections).

^c^ All continuous outcomes were standardized (mean=0, standard deviation=1), and β was the standardized effect size.

^d^ p<0.05 after Bonferroni correction (the p value cutoff for Bonferroni correction is p=0.05/28 outcomes=0.002; the models for the outcomes of community engagement and PTSD did not converge, thus these outcomes were not included in the table).
