# Supplementary material for: School types in adolescence and subsequent health and well-being in young adulthood: An outcome-wide analysis
Source: PLoS One. 2021 Nov 10;16(11):e0258723. doi: 10.1371/journal.pone.0258723 (PMC8580227; doi:10.1371/journal.pone.0258723)
Supplement: S3 Table — A. School type in adolescence and subsequent health and well-being in young adulthood, among those residing in low socioeconomic status neighborhoods. B. School type in adolescence and subsequent health and well-being in young adulthood, among those residing in high socioeconomic status neighborhoods. (DOCX) [file pone.0258723.s003.docx]

**S3A Table. School type in adolescence and subsequent health and well-being in young adulthood, among those residing in low socioeconomic status neighborhoods ^a^ (Growing Up Today Study from 1999 to 2007, 2010 or 2013 questionnaire wave, N=8,845)**

|  | **School Types** ^b^ | | | | | | | | |
| --- | --- | --- | --- | --- | --- | --- | --- | --- | --- |
|  | Private school vs. public school | | | |  | Religious school vs. public school | | | |
| Health and well-being outcomes | RR | β ^c^ | 95% CI | P-value ^d^ |  | RR | β ^c^ | 95% CI | P-value ^d^ |
| **Psychological Well-being** |  |  |  |  |  |  |  |  |  |
| Life satisfaction |  | -0.01 | -0.10, 0.09 | 0.90 |  |  | 0.04 | -0.06, 0.13 | 0.47 |
| Positive affect |  | 0.02 | -0.07, 0.10 | 0.65 |  |  | 0.04 | -0.05, 0.13 | 0.43 |
| Self-esteem |  | 0.01 | -0.07, 0.10 | 0.76 |  |  | -0.03 | -0.12, 0.05 | 0.44 |
| Emotional processing |  | -0.03 | -0.13, 0.06 | 0.46 |  |  | 0.01 | -0.08, 0.09 | 0.84 |
| Emotional expression |  | 0.02 | -0.08, 0.11 | 0.74 |  |  | 0.04 | -0.05, 0.12 | 0.42 |
| **Social Engagement** |  |  |  |  |  |  |  |  |  |
| Being married | 0.97 |  | 0.86, 1.08 | 0.53 |  | 0.92 |  | 0.83, 1.02 | 0.12 |
| Community engagement | 0.97 |  | 0.88, 1.06 | 0.52 |  | 0.97 |  | 0.88, 1.06 | 0.47 |
| Educational attainment (≥college) | 0.97 |  | 0.92, 1.02 | 0.25 |  | 0.98 |  | 0.94, 1.03 | 0.41 |
| **Character Strengths** |  |  |  |  |  |  |  |  |  |
| Frequency of volunteering |  | 0.00 | -0.08, 0.09 | 0.92 |  |  | -0.02 | -0.11, 0.07 | 0.66 |
| Sense of mission |  | -0.02 | -0.11, 0.07 | 0.68 |  |  | -0.02 | -0.11, 0.07 | 0.65 |
| Forgiveness of others |  | **0.09** | **0.00, 0.17** | **0.04** |  |  | 0.07 | -0.02, 0.16 | 0.11 |
| Registered to vote | 1.00 |  | 0.97, 1.02 | 0.78 |  | 1.03 |  | 1.00, 1.06 | 0.08 |
| **Mental Health** |  |  |  |  |  |  |  |  |  |
| Depressive symptoms |  | 0.01 | -0.09, 0.10 | 0.90 |  |  | 0.02 | -0.07, 0.11 | 0.68 |
| Depression diagnosis | 0.95 |  | 0.76, 1.19 | 0.64 |  | 0.98 |  | 0.81, 1.18 | 0.83 |
| Anxiety symptoms |  | 0.00 | -0.08, 0.08 | 0.92 |  |  | 0.06 | -0.02, 0.15 | 0.13 |
| Anxiety diagnosis | 1.00 |  | 0.79, 1.25 | 0.97 |  | 1.00 |  | 0.80, 1.26 | 0.98 |
| **Health Behaviors** |  |  |  |  |  |  |  |  |  |
| Current cigarette smoking | 1.07 |  | 0.94, 1.22 | 0.28 |  | 1.07 |  | 0.92, 1.24 | 0.39 |
| Frequent binge drinking | 1.04 |  | 0.93, 1.18 | 0.47 |  | **1.13** |  | **1.01, 1.28** | **0.04** |
| Marijuana use | 1.00 |  | 0.91, 1.09 | 0.93 |  | 1.02 |  | 0.94, 1.10 | 0.65 |
| Any other illicit drug use | 0.98 |  | 0.84, 1.13 | 0.75 |  | 1.04 |  | 0.88, 1.23 | 0.62 |
| Prescription drug misuse | 0.98 |  | 0.84, 1.16 | 0.85 |  | 0.93 |  | 0.78, 1.11 | 0.42 |
| Number of lifetime sexual partners |  | -0.06 | -0.14, 0.01 | 0.11 |  |  | **-0.09** | **-0.16, -0.02** | **0.01** |
| History of STIs | 0.86 |  | 0.67, 1.09 | 0.21 |  | 0.96 |  | 0.76, 1.21 | 0.72 |
| Preventive healthcare use | 0.94 |  | 0.87, 1.02 | 0.16 |  | 0.98 |  | 0.90, 1.06 | 0.53 |
| **Physical Health** |  |  |  |  |  |  |  |  |  |
| Overweight/obesity | 0.95 |  | 0.86, 1.04 | 0.25 |  | **0.89** |  | **0.80, 0.99** | **0.03** |
| No. of physical health problems |  | -0.01 | -0.09, 0.08 | 0.90 |  |  | -0.01 | -0.10, 0.08 | 0.86 |

Abbreviations: RR, risk ratio; CI, confidence interval.

^a^ The full analytic sample was restricted to those who responded to the Growing Up Today Study 1999 questionnaire wave in which the exposure school type was assessed, and was also restricted to those who resided in a neighborhood with a census-tract median income <$75,000. Multiple imputation was performed to impute missing data on all variables. Participants who were home schooled were excluded from this analysis due to the small sample size. In the analytic sample, the sample size for each school type was 7,160 for public school, 877 for private school, and 808 for religious school.

^b^ A set of generalized estimating equations were used to regress each outcome on school type separately. All models controlled for participants’ age, sex, race/ethnicity, puberty development, geographic region, mother’s age, mother’s race/ethnicity, mother’s marital status, socioeconomic status (including mother’s subjective socioeconomic status, mother’s employment status, father’s educational attainment, household income, and census-tract college education rate), participant family environment (including family structure, family dinner frequency, maternal relationship satisfaction, frequency of religious service attendance, maternal depression, and maternal smoking), and participant prior health status or prior health behaviors (prior depressive symptoms, overweight/obesity, smoking, drinking, marijuana use, other drug use, prescription, drug misuse, number of sexual partners, early sexual initiation, and history of sexually transmitted infections).

^c^ All continuous outcomes were standardized (mean=0, standard deviation=1), and β was the standardized effect size.

^d^ There was no association that reached p<.05 after Bonferroni correction in this table (the model for the outcome short sleep duration, PTSD, religious service attendance and early sexual initiation did not converge, thus these outcomes were not included in the table).

**S3B Table. School type in adolescence and subsequent health and well-being in young adulthood, among those residing in high socioeconomic status neighborhoods ^a^ (Growing Up Today Study from 1999 to 2007, 2010 or 2013 questionnaire wave, N=820)**

|  | **School Types** ^b^ | | | | | | | | |
| --- | --- | --- | --- | --- | --- | --- | --- | --- | --- |
|  | Private school vs. public school | | | |  | Religious school vs. public school | | | |
| Health and well-being outcomes | RR | β ^c^ | 95% CI | P-value ^d^ |  | RR | β ^c^ | 95% CI | P-value ^d^ |
| **Psychological Well-being** |  |  |  |  |  |  |  |  |  |
| Life satisfaction |  | 0.06 | -0.19, 0.32 | 0.62 |  |  | 0.09 | -0.20, 0.38 | 0.54 |
| Positive affect |  | 0.12 | -0.14, 0.38 | 0.38 |  |  | 0.13 | -0.17, 0.43 | 0.39 |
| Self-esteem |  | 0.12 | -0.11, 0.34 | 0.30 |  |  | 0.06 | -0.25, 0.37 | 0.69 |
| Emotional processing |  | 0.09 | -0.15, 0.33 | 0.47 |  |  | 0.02 | -0.25, 0.30 | 0.87 |
| Emotional expression |  | -0.03 | -0.29, 0.23 | 0.83 |  |  | -0.01 | -0.26, 0.25 | 0.97 |
| **Social Engagement** |  |  |  |  |  |  |  |  |  |
| Being married | 0.83 |  | 0.54, 1.27 | 0.39 |  | 1.00 |  | 0.67, 1.51 | 0.98 |
| Community engagement | 0.84 |  | 0.63, 1.12 | 0.24 |  | 0.91 |  | 0.65, 1.26 | 0.56 |
| Educational attainment (≥college) | 0.99 |  | 0.91, 1.09 | 0.86 |  | 1.00 |  | 0.90, 1.12 | 0.96 |
| **Character Strengths** |  |  |  |  |  |  |  |  |  |
| Frequency of volunteering |  | 0.04 | -0.18, 0.26 | 0.73 |  |  | -0.12 | -0.37, 0.12 | 0.33 |
| Sense of mission |  | 0.10 | -0.11, 0.32 | 0.35 |  |  | -0.11 | -0.36, 0.14 | 0.38 |
| Forgiveness of others |  | 0.17 | -0.05, 0.40 | 0.13 |  |  | 0.00 | -0.25, 0.25 | 0.99 |
| Registered to vote | 1.00 |  | 0.93, 1.08 | 0.95 |  | 1.00 |  | 0.93, 1.09 | 0.92 |
| **Mental Health** |  |  |  |  |  |  |  |  |  |
| Depressive symptoms |  | 0.00 | -0.26, 0.25 | 0.97 |  |  | -0.07 | -0.33, 0.19 | 0.60 |
| Depression diagnosis | 0.89 |  | 0.52, 1.53 | 0.68 |  | 0.79 |  | 0.43, 1.44 | 0.44 |
| Anxiety symptoms |  | -0.04 | -0.31, 0.22 | 0.74 |  |  | -0.02 | -0.31, 0.28 | 0.92 |
| Anxiety diagnosis | 0.75 |  | 0.38, 1.46 | 0.39 |  | 0.74 |  | 0.31, 1.77 | 0.50 |
| **Health Behaviors** |  |  |  |  |  |  |  |  |  |
| Current cigarette smoking | 1.20 |  | 0.83, 1.73 | 0.33 |  | 1.12 |  | 0.72, 1.74 | 0.63 |
| Frequent binge drinking | 1.24 |  | 0.94, 1.63 | 0.13 |  | 1.25 |  | 0.91, 1.72 | 0.17 |
| Marijuana use | 1.04 |  | 0.89, 1.23 | 0.61 |  | 1.00 |  | 0.83, 1.21 | 0.99 |
| Any other illicit drug use | 1.29 |  | 0.93, 1.77 | 0.13 |  | 1.01 |  | 0.64, 1.60 | 0.96 |
| Prescription drug misuse | 0.99 |  | 0.72, 1.36 | 0.97 |  | 0.99 |  | 0.65, 1.51 | 0.97 |
| Number of lifetime sexual partners |  | 0.10 | -0.13, 0.32 | 0.40 |  |  | 0.01 | -0.26, 0.28 | 0.92 |
| History of STIs | 0.86 |  | 0.45, 1.64 | 0.65 |  | 0.80 |  | 0.33, 1.98 | 0.63 |
| Preventive healthcare use | 0.98 |  | 0.78, 1.23 | 0.86 |  | 0.97 |  | 0.75, 1.26 | 0.83 |
| **Physical Health** |  |  |  |  |  |  |  |  |  |
| Overweight/obesity | 1.07 |  | 0.78, 1.48 | 0.67 |  | 0.87 |  | 0.58, 1.32 | 0.52 |
| No. of physical health problems |  | 0.14 | -0.11, 0.40 | 0.27 |  |  | -0.09 | -0.34, 0.16 | 0.48 |

Abbreviations: RR, risk ratio; CI, confidence interval.

^a^ The full analytic sample was restricted to those who responded to the Growing Up Today Study 1999 questionnaire wave in which the exposure school type was assessed, and was also restricted to those who resided in a neighborhood with a census-tract median income ≥$100,000. Multiple imputation was performed to impute missing data on all variables. Participants who were home schooled were excluded from this analysis due to the small sample size. In the analytic sample, the sample size for each school type was 622 for public school, 118 for private school, and 80 for religious school.

^b^ A set of generalized estimating equations were used to regress each outcome on school type separately. All models controlled for participants’ age, sex, race/ethnicity, puberty development, geographic region, mother’s age, mother’s race/ethnicity, mother’s marital status, socioeconomic status (including mother’s subjective socioeconomic status, mother’s employment status, father’s educational attainment, household income, and census-tract college education rate), participant family environment (including family structure, family dinner frequency, maternal relationship satisfaction, frequency of religious service attendance, maternal depression, and maternal smoking), and participant prior health status or prior health behaviors (prior depressive symptoms, overweight/obesity, smoking, drinking, marijuana use, other drug use, prescription, drug misuse, number of sexual partners, early sexual initiation, and history of sexually transmitted infections).

^c^ All continuous outcomes were standardized (mean=0, standard deviation=1), and β was the standardized effect size.

^d^ There was no association that reached p<.05 either before or after Bonferroni correction in this table.
