# Supplementary material for: School types in adolescence and subsequent health and well-being in young adulthood: An outcome-wide analysis
Source: PLoS One. 2021 Nov 10;16(11):e0258723. doi: 10.1371/journal.pone.0258723 (PMC8580227; doi:10.1371/journal.pone.0258723)
Supplement: S4 Table — A. School type in adolescence and subsequent health and well-being in young adulthood, among those residing in the top 10 states with the lowest public school ranking. B. School type in adolescence and subsequent health and well-being in young adulthood, among those residing in the top 10 states with the highest public school ranking. (DOCX) [file pone.0258723.s004.docx]

**S4A Table. School type in adolescence and subsequent health and well-being in young adulthood, among those residing in the top 10 states with the lowest public school ranking ^a^ (Growing Up Today Study from 1999 to 2007, 2010 or 2013 questionnaire wave, N=237)**

|  | **School Types** ^b^ | | | | | | |
| --- | --- | --- | --- | --- | --- | --- | --- |
|  | Private school vs. public school | | |  | Religious school vs. public school | | |
| Health and well-being outcomes | β ^c^ | 95% CI | P-value ^d^ |  | β ^c^ | 95% CI | P-value ^d^ |
| **Psychological Well-being** |  |  |  |  |  |  |  |
| Life satisfaction | 0.01 | -0.49, 0.51 | 0.97 |  | 0.32 | -0.18, 0.83 | 0.21 |
| Positive affect | 0.02 | -0.44, 0.48 | 0.93 |  | 0.35 | -0.20, 0.90 | 0.21 |
| Self-esteem | 0.19 | -0.29, 0.67 | 0.43 |  | 0.34 | -0.18, 0.87 | 0.19 |
| Emotional processing | -0.18 | -0.66, 0.29 | 0.45 |  | 0.12 | -0.38, 0.62 | 0.63 |
| Emotional expression | 0.16 | -0.34, 0.66 | 0.53 |  | 0.33 | -0.19, 0.86 | 0.21 |
| **Character Strengths** |  |  |  |  |  |  |  |
| Frequency of volunteering | -0.08 | -0.52, 0.36 | 0.72 |  | -0.33 | -0.81, 0.16 | 0.19 |
| Sense of mission | -0.17 | -0.66, 0.32 | 0.50 |  | -0.25 | -0.75, 0.26 | 0.34 |
| Forgiveness of others | -0.12 | -0.58, 0.34 | 0.60 |  | 0.10 | -0.43, 0.63 | 0.71 |
| **Mental Health** |  |  |  |  |  |  |  |
| Depressive symptoms | -0.11 | -0.61, 0.38 | 0.65 |  | -0.22 | -0.75, 0.32 | 0.43 |
| Anxiety symptoms | -0.27 | -0.75, 0.20 | 0.26 |  | -0.29 | -0.83, 0.25 | 0.30 |
| **Health Behaviors** |  |  |  |  |  |  |  |
| Number of lifetime sexual partners | 0.30 | -0.17, 0.76 | 0.21 |  | 0.04 | -0.46, 0.55 | 0.86 |
| **Physical Health** |  |  |  |  |  |  |  |
| No. of physical health problems | -0.27 | -0.74, 0.20 | 0.26 |  | -0.43 | -0.95, 0.10 | 0.11 |

Abbreviations: CI, confidence interval.

^a^ The analytic sample was restricted to those who responded to the Growing Up Today Study 1999 questionnaire wave in which the exposure school type was assessed, and was also restricted to those who resided in the top 10 states with the lowest public school ranking (NM, AL, NV, AK, LA, MS, AZ, OK, WV, SC). Multiple imputation was performed to impute missing data on all variables. Participants who were home schooled were excluded from this analysis due to the small sample size. In this sample, the sample size for each school type was 184 for public school, 33 for private school, and 20 for religious school.

^b^ A set of generalized estimating equations were used to regress each outcome on school type separately. All models controlled for participants’ age, sex, race/ethnicity, puberty development, mother’s age, mother’s race/ethnicity, mother’s marital status, socioeconomic status (including mother’s subjective socioeconomic status, mother’s employment status, father’s educational attainment, household income, census tract college education rate, and census tract median income), participant family environment (including family structure, family dinner frequency, maternal relationship satisfaction, religious service attendance, maternal depression, and maternal smoking), and participant prior health status or prior health behaviors (prior depressive symptoms, overweight/obesity, smoking, drinking, marijuana use, other drug use, prescription, drug misuse, number of sexual partners, early sexual initiation, and history of sexually transmitted infections).

^c^ All continuous outcomes were standardized (mean=0, standard deviation=1), and β was the standardized effect size.

^d^ There was no association that reached p<.05 either before or after Bonferroni correction in this table (the model for the outcome depression diagnosis, anxiety diagnosis, educational attainment, marital status, religious service attendance, community engagement, voting registration, short sleep duration, PTSD, smoking, binge drinking, marijuana use, other illicit drug use, prescription drug misuse, early sexual initiation, history of STIs, preventive healthcare use and overweight/obesity did not converge, thus these outcomes were not included in this table).

**S4B Table. School type in adolescence and subsequent health and well-being in young adulthood, among those residing in the top 10 states with the highest public school ranking^a^ (Growing Up Today Study from 1999 to 2007, 2010 or 2013 questionnaire wave, N=3,120)**

|  | **School Types** ^b^ | | | | | | | | |
| --- | --- | --- | --- | --- | --- | --- | --- | --- | --- |
|  | Private school vs. public school | | | |  | Religious school vs. public school | | | |
| Health and well-being outcomes | RR | β ^c^ | 95% CI | P-value ^d^ |  | RR | β ^c^ | 95% CI | P-value ^d^ |
| **Psychological Well-being** |  |  |  |  |  |  |  |  |  |
| Life satisfaction |  | -0.02 | -0.16, 0.12 | 0.78 |  |  | 0.03 | -0.11, 0.16 | 0.70 |
| Positive affect |  | 0.00 | -0.14, 0.15 | 0.98 |  |  | 0.09 | -0.06, 0.24 | 0.23 |
| Self-esteem |  | 0.01 | -0.12, 0.15 | 0.84 |  |  | -0.03 | -0.16, 0.10 | 0.65 |
| Emotional processing |  | -0.01 | -0.15, 0.14 | 0.93 |  |  | 0.00 | -0.14, 0.14 | 0.98 |
| Emotional expression |  | -0.04 | -0.22, 0.13 | 0.61 |  |  | 0.07 | -0.08, 0.23 | 0.35 |
| **Social Engagement** |  |  |  |  |  |  |  |  |  |
| Being married | 0.94 |  | 0.75, 1.16 | 0.54 |  | 0.85 |  | 0.70, 1.03 | 0.10 |
| Religious service attendance  (≥ once per week) | 1.22 |  | 0.95, 1.58 | 0.12 |  | 1.26 |  | 0.98, 1.63 | 0.07 |
| **Character Strengths** |  |  |  |  |  |  |  |  |  |
| Frequency of volunteering |  | 0.08 | -0.07, 0.23 | 0.29 |  |  | -0.02 | -0.17, 0.12 | 0.74 |
| Sense of mission |  | -0.05 | -0.20, 0.10 | 0.52 |  |  | 0.06 | -0.08, 0.19 | 0.39 |
| Forgiveness of others |  | 0.06 | -0.08, 0.21 | 0.39 |  |  | 0.05 | -0.08, 0.18 | 0.45 |
| Registered to vote | 0.98 |  | 0.93, 1.05 | 0.62 |  | 1.03 |  | 0.98, 1.08 | 0.30 |
| **Mental Health** |  |  |  |  |  |  |  |  |  |
| Depressive symptoms |  | 0.08 | -0.08, 0.24 | 0.34 |  |  | 0.00 | -0.14, 0.15 | 0.95 |
| Depression diagnosis | 0.92 |  | 0.60, 1.40 | 0.69 |  | 1.10 |  | 0.79, 1.52 | 0.58 |
| Anxiety symptoms |  | 0.03 | -0.13, 0.18 | 0.74 |  |  | 0.07 | -0.08, 0.21 | 0.36 |
| Anxiety diagnosis | 1.01 |  | 0.73, 1.39 | 0.96 |  | 1.17 |  | 0.85, 1.61 | 0.34 |
| **Health Behaviors** |  |  |  |  |  |  |  |  |  |
| Current cigarette smoking | 1.05 |  | 0.81, 1.36 | 0.70 |  | 1.03 |  | 0.80, 1.33 | 0.81 |
| Marijuana use | 0.97 |  | 0.84, 1.12 | 0.67 |  | 1.01 |  | 0.88, 1.16 | 0.91 |
| Number of lifetime sexual partners |  | -0.09 | -0.22, 0.05 | 0.21 |  |  | -0.02 | -0.15, 0.11 | 0.75 |
| Early sexual initiation | 0.89 |  | 0.57, 1.41 | 0.62 |  | 0.86 |  | 0.58, 1.29 | 0.47 |
| History of STIs | 0.76 |  | 0.48, 1.19 | 0.22 |  | 1.04 |  | 0.73, 1.48 | 0.83 |
| Preventive healthcare use | 0.86 |  | 0.74, 1.01 | 0.07 |  | 0.98 |  | 0.86, 1.12 | 0.79 |
| **Physical Health** |  |  |  |  |  |  |  |  |  |
| Overweight/obesity | 0.95 |  | 0.79, 1.13 | 0.54 |  | 0.91 |  | 0.75, 1.10 | 0.33 |
| No. of physical health problems |  | 0.00 | -0.16, 0.16 | 0.96 |  |  | -0.02 | -0.16, 0.13 | 0.81 |

Abbreviations: RR, risk ratio; CI, confidence interval.

^a^ The full analytic sample was restricted to those who responded to the Growing Up Today Study 1999 questionnaire wave in which the exposure school type was assessed, and was also restricted to those who resided in the top 10 states with the lowest public school ranking (MA, NJ, NH, VT, CT, IN, IL, VA, NE, PA). Multiple imputation was performed to impute missing data on all variables. Participants who were home schooled were excluded from this analysis due to the small sample size. In the analytic sample, the sample size for each school type was 2,530 for public school, 298 for private school, and 292 for religious school.

^b^ A set of generalized estimating equations were used to regress each outcome on school type separately. All models controlled for participants’ age, sex, race/ethnicity, puberty development, mother’s age, mother’s race/ethnicity, mother’s marital status, socioeconomic status (including mother’s subjective socioeconomic status, mother’s employment status, father’s educational attainment, household income, census tract college education rate, and census tract median income), participant family environment (including family structure, family dinner frequency, maternal relationship satisfaction, frequency of religious service attendance, maternal depression, and maternal smoking), and participant prior health status or prior health behaviors (prior depressive symptoms, overweight/obesity, smoking, drinking, marijuana use, other drug use, prescription, drug misuse, number of sexual partners, early sexual initiation, and history of sexually transmitted infections).

^c^ All continuous outcomes were standardized (mean=0, standard deviation=1), and β was the standardized effect size.

^d^ There was no association that reached p<.05 either before or after Bonferroni correction in this table (the model for the outcome binge drinking, other illicit drug use, prescription drug misuse, educational attainment, community engagement, short sleep duration, and PTSD did not converge, thus these outcomes were not included in the table).
