# Supplementary material for: School types in adolescence and subsequent health and well-being in young adulthood: An outcome-wide analysis
Source: PLoS One. 2021 Nov 10;16(11):e0258723. doi: 10.1371/journal.pone.0258723 (PMC8580227; doi:10.1371/journal.pone.0258723)
Supplement: S5 Table — (DOCX) [file pone.0258723.s005.docx]

**S5 Table. Attending religious schools versus public schools in adolescence and subsequent health and well-being in young adulthood, stratified by frequency of religious service attendance in adolescence (Growing Up Today Study from 1999 to 2007, 2010 or 2013 questionnaire wave, N=10,821^a^)**

|  | **Religious School vs. Public School** ^b^ | | | | | | | | |
| --- | --- | --- | --- | --- | --- | --- | --- | --- | --- |
|  | **< 1/week religious service attendance (n=4,971)** | | | |  | **≥ 1/week religious service attendance (n=5,850)** | | | |
| Health and well-being outcomes | RR | β ^c^ | 95% CI | P-value^d^ |  | RR | β ^c^ | 95% CI | P-value^d^ |
| **Psychological Well-being** |  |  |  |  |  |  |  |  |  |
| Life satisfaction |  | 0.10 | -0.06, 0.26 | 0.22 |  |  | 0.05 | -0.04, 0.14 | 0.29 |
| Positive affect |  | 0.10 | -0.04, 0.25 | 0.17 |  |  | 0.05 | -0.04, 0.15 | 0.24 |
| Self-esteem |  | 0.01 | -0.14, 0.16 | 0.92 |  |  | -0.02 | -0.11, 0.06 | 0.60 |
| Emotional processing |  | 0.04 | -0.10, 0.19 | 0.57 |  |  | 0.00 | -0.09, 0.08 | 0.98 |
| Emotional expression |  | 0.04 | -0.12, 0.19 | 0.64 |  |  | 0.03 | -0.05, 0.12 | 0.47 |
| **Social Engagement** |  |  |  |  |  |  |  |  |  |
| Being married | 0.94 |  | 0.78, 1.15 | 0.56 |  | 0.92 |  | 0.83, 1.03 | 0.14 |
| Community engagement | 0.98 |  | 0.82, 1.16 | 0.79 |  | 1.01 |  | 0.92, 1.11 | 0.84 |
| Educational attainment (≥college) | 1.00 |  | 0.93, 1.08 | 0.93 |  | 0.99 |  | 0.94, 1.03 | 0.51 |
| **Character Strengths** |  |  |  |  |  |  |  |  |  |
| Frequency of volunteering |  | 0.08 | -0.07, 0.23 | 0.28 |  |  | -0.04 | -0.13, 0.04 | 0.32 |
| Sense of mission |  | -0.01 | -0.16, 0.14 | 0.87 |  |  | -0.01 | -0.10, 0.07 | 0.77 |
| Forgiveness of others |  | 0.14 | -0.01, 0.29 | 0.06 |  |  | 0.07 | -0.01, 0.15 | 0.10 |
| Registered to vote | 1.02 |  | 0.97, 1.08 | 0.39 |  | **1.03** |  | **1.00, 1.05** | **0.04** |
| **Mental Health** |  |  |  |  |  |  |  |  |  |
| Depressive symptoms |  | -0.03 | -0.19, 0.13 | 0.73 |  |  | 0.02 | -0.08, 0.11 | 0.73 |
| Depression diagnosis | 0.88 |  | 0.64, 1.22 | 0.44 |  | 0.97 |  | 0.80, 1.17 | 0.76 |
| Anxiety symptoms |  | -0.04 | -0.18, 0.10 | 0.56 |  |  | 0.07 | -0.02, 0.16 | 0.12 |
| Anxiety diagnosis | 0.80 |  | 0.53, 1.22 | 0.31 |  | 0.99 |  | 0.79, 1.25 | 0.96 |
| **Health Behaviors** |  |  |  |  |  |  |  |  |  |
| Current cigarette smoking | 1.08 |  | 0.88, 1.33 | 0.47 |  | 1.14 |  | 0.97, 1.33 | 0.10 |
| Frequent binge drinking | 1.09 |  | 0.89, 1.34 | 0.42 |  | **1.17** |  | **1.04, 1.31** | **0.01** |
| Marijuana use | 0.97 |  | 0.87, 1.08 | 0.61 |  | 1.03 |  | 0.96, 1.12 | 0.39 |
| Any other illicit drug use | 0.97 |  | 0.79, 1.20 | 0.79 |  | 1.04 |  | 0.88, 1.23 | 0.66 |
| Number of lifetime sexual partners |  | -0.09 | -0.21, 0.03 | 0.16 |  |  | **-0.08** | **-0.15, -0.01** | **0.03** |
| Early sexual initiation | 1.05 |  | 0.77, 1.44 | 0.74 |  | 0.84 |  | 0.62, 1.15 | 0.27 |
| Preventive healthcare use | 0.96 |  | 0.83, 1.12 | 0.64 |  | 0.99 |  | 0.92, 1.06 | 0.70 |
| **Physical Health** |  |  |  |  |  |  |  |  |  |
| Overweight/obesity | 0.97 |  | 0.79, 1.18 | 0.75 |  | **0.89** |  | **0.80, 0.99** | **0.04** |
| No. of physical health problems |  | 0.01 | -0.15, 0.18 | 0.88 |  |  | -0.01 | -0.09, 0.06 | 0.71 |

Abbreviations: RR, risk ratio; CI, confidence interval.

^a^ The full analytic sample was restricted to those who responded to the Growing Up Today Study 1999 questionnaire wave in which the exposure school type was assessed. Multiple imputation was performed to impute missing data on all variables.

^b^ A set of generalized estimating equations were used to regress each outcome on school type separately. All models controlled for participants’ age, sex, race/ethnicity, puberty development, geographic region, mother’s age, mother’s race/ethnicity, mother’s marital status, socioeconomic status (including mother’s subjective socioeconomic status, mother’s employment status, father’s educational attainment, household income, census tract college education rate, and census tract median income), participant family environment (including family structure, family dinner frequency, maternal relationship satisfaction, maternal depression, and maternal smoking), and participant prior health status or prior health behaviors (prior depressive symptoms, overweight/obesity, smoking, drinking, marijuana use, other drug use, prescription, drug misuse, number of sexual partners, early sexual initiation, and history of sexually transmitted infections).

^c^ All continuous outcomes were standardized (mean=0, standard deviation=1), and β was the standardized effect size.

^d^ There was no association that reached p<.05 after Bonferroni correction in this table (the models for the outcomes of religious service attendance, probable PTSD, prescription drug use, STIs and short sleep duration did not converge, thus these outcomes were not included in this table).
