# Supplementary material for: School types in adolescence and subsequent health and well-being in young adulthood: An outcome-wide analysis
Source: PLoS One. 2021 Nov 10;16(11):e0258723. doi: 10.1371/journal.pone.0258723 (PMC8580227; doi:10.1371/journal.pone.0258723)
Supplement: S1 Text — (DOCX) [file pone.0258723.s007.docx]

**Supplementary Text**

Assessment of outcome variables

Psychological Well-being

*Life satisfaction*. One item from the validated Rand Mental Health Inventory [1] (i.e., “Have you felt happy, satisfied or please with your personal life”) was used to measure life satisfaction over the past month (wave 2010). Response categories ranged from 1: none of the time to 6: all of the time. The response was used as a continuous variable.

*Positive affect*. Positive affect over the past month (wave 2010) was measured with 10 items from the previously validated Rand Mental Health Inventory [1] (e.g., “Have you felt cheerful, lighthearted”). Response categories ranged from 1 (none of the time) to 6 (all of the time). An overall score was created by summing responses across all items (α = 0.91), ranging from 10 to 60.

*Self-esteem.* Self-esteem was measured with the validated Rosenberg Self-esteem Scale [2, 3], which assessed global self-worth (wave 2010). The scale included questions on both positive (e.g., “I feel that I have a number of good qualities”) and negative feelings (e.g., “I feel I do not have much to be proud of”) towards oneself. Response categories ranged from 1 (strongly agree) to 4 (strongly disagree). Responses on positive feelings were reverse coded so that higher scores reflected greater self-esteem. An overall score was calculated by summing responses across all items (α = 0.91), ranging from 10 to 40.

*Emotional processing*. Emotional processing was measured with the 4-item emotional processing subscale from the validated Emotional Approach Coping Scale[4] (wave 2010). The extent to which participants acknowledge and understand their emotions in stressful situations were assessed (e.g, “I take time to figure out what I am really feeling”). Response categories ranged from 1 (not at all) to 4 (a lot). An overall score was calculated by summing responses across all items (α = 0.84), ranging from 4 to 16.

*Emotional expression*. Emotional expression was measured with the 4-item emotional expression subscale from the validated Emotional Approach Coping Scale [4] (wave 2010). The subscale assessed the extent to which participants express their emotions under stress (e.g, “I let my feelings come out freely”). Response categories ranged from 1: not at all to 4: a lot. An overall score was created by summing responses across all items (α = 0.91), ranging from 4 to 16.
Social Engagement

*Being married*. Participants were asked to report their current marital status (wave 2010). Response options included never married, married, living with partner, separated, divorced and widowed. Those who reported being married or living with partner were considered as being married, whereas other options were collapsed as not being married.

*Community engagement*. One item was used to assess community engagement: “In the past year, how often have you donated time to a community or neighborhood organization” (wave 2010). Response options included never, 1 time, 2-5 times, 6-11 times, and 12+ times. The responses of ≥1 time were collapsed to create a dichotomized measure of community engagement (never, ever).

*Religious service attendance*. Participants reported their attendance at religious services in response to the question: “How often do you go to religious meetings or services” (wave 07). The responses included never, less than once a month, 1-3 times per month, once a week, and more than once a week. The top two levels were collapsed and the lower three categories were combined, to create an indicator of at least once/week attendance at religious services.

*Educational attainment*. Participants reported their educational attainment in response to the question: “What is the highest grade of school you have completed or the highest degree you have received” (wave 10). Participants who reported associate degree (2-year college), bachelor’s degree, master’s degree, and doctoral degree were considered as having a college or higher degree. Those who reported some higher school, high school graduate or the equivalent, trade/vocational school certificate/diploma, and some college were considered as not having a college degree.

Character Strengths

*Frequency of volunteering*. Participants reported their frequency of volunteering in response to the question: “In an average month, how many hours do you spend on volunteer work, community service, or helping people outside of your home without getting paid” (wave 07). Response options ranged from 1 (0 hour) to 4 (10 or more hours). The response was used as a continuous variable.

*Sense of mission*. Participants reported to what extent they agreed with the statement: “I have a sense of mission or calling in my own life” (wave 07). Response ranged from 1 (strongly agree) to 4 (strongly disagree). The response was reverse coded so that higher scores reflected greater sense of mission. The response was used as a continuous variable.

*Forgiveness of others*. Forgiveness was assessed with a single question: “Because of my spiritual or religious beliefs, I have forgiven those who hurt me” (wave 07). Response categories ranged from 1 (always or almost always) to 4 (never). Responses were reverse coded so that higher scores reflected greater forgiveness. The response was considered as a continuous score.

*Voting registration status*. Participants reported whether they were currently registered to vote (yes, no) (wave 2007).

Mental Health

*Depression.* Depressive symptoms (wave 2010) was measured with the validated 10-item Center for Epidemiologic Studies Depression Scale (CES-D) [5] which assessed participants’ feelings over the past week (e.g., “I felt depressed”; “I was happy”). Response categories ranged from 0 (rarely or none of the time) to 3 (all of the time). Responses to the positive items were reverse coded so that a higher score reflected more depressive symptoms. An overall score was created by summing responses across all items (α =0.81), ranging from 0 to 30. In addition, participants also reported whether they had been told by a health care provider that they had depression (wave 2013). Those who responded affirmatively were considered as having depression diagnosis.

*Anxiety.* Anxiety symptoms (wave 2010) was measured with 9 items (e.g., “I worry a lot of the time”) from the Worry/Sensitivity Subscale of the Revised Children’s Manifest Anxiety Scale (RCMAS) which had been well-validated in youth [6-8]. Response categories ranged from 0 (none of the time) to 5 (all of the time). An overall score was created by summing responses across all items (α = 0.93), ranging from 9 to 54. In addition, participants also reported whether they had been told by a health care provider that they had anxiety disorder (wave 2013). Those who responded affirmatively were considered as having anxiety diagnosis.

*Probable post-traumatic stress disorder (PTSD).* Lifetime PTSD symptoms (wave 2007) was assessed with Breslau’s 7-item short screening scale which had been validated in young adults [9]. Specifically, participants were asked to think about the most distressing event in their lifetime, and report symptoms of PTSD in reference to the event. Those who reported presence of 6 or more symptoms were considered as having probable PTSD [9, 10].

Health Behaviors

*Cigarette smoking*. In GUTS2 2013 wave, participants were asked to report their frequency of smoking in response to the question: “In the past 12 months, how often did you smoke cigarettes”. The response categories ranged from 1 (never) to 5 (daily). The responses were dichotomized as ever (2: less than once a month to 5: daily) and never smoking (1: never).

*Frequent binge drinking*. Binge drinking over the past 12 months was assessed with a single question (wave 2010): “In the past 12 months, how many times did you drink 5 (for male)/4(for female) or more alcoholic drinks over a few hours”. Categorical response options ranged from 1 (never) to 10 (37 or more times). Participants who reported at least 12 episodes of binge drinking (7: 12 to 15 times to 10: 37 or more times) were considered as frequent binge drinkers [11].

*Marijuana use*. Participants reported their marijuana use over the past 12 months (wave 2010): “In the past 12 months, how many times did you use marijuana”. Response categories ranged from 1 (never) to 6 (6 or more times/week). Responses were dichotomized as ever (2: once a month or less to 6: six or more times per week) and never (1: never) use of marijuana.

*Other Illicit drug use.* Participants reported their use of the following drugs in past 12 months: cocaine or crack, heroin, ecstasy, LSD/mushrooms or any other hallucinogen, crystal meth, or other amphetamines (wave 2010). Categorical response options ranged from 1 (not in the past 12 months) to 6 (16 or more times). Responses were dichotomized as never (1: not in the past 12 months) and ever use (2: 1 time to 6: 16 or more times) for each drug separately. Those who reported use of any of these drugs were considered as having illicit drug use other than marijuana.

*Prescription drug misuse*. Participants reported their use of the following drugs without a doctor’s prescription over the past 12 months: tranquilizers, pain killers, sleeping pills and stimulants (GUTS1 2010). Categorical response options ranged from 1 (not in the past 12 months) to 6 (16 or more times). Responses were dichotomized as never (1: not in the past 12 months) and ever use (2: 1 time to 6: 16 or more times) for each drug separately. Those who reported use of any of these drugs were considered as having prescription drug misuse.

*Number of lifetime sexual partners*. Participants were first asked whether they ever had sexual intercourse (yes, no, not sure). Those who answered “yes” or “not sure” were further asked to report the number of their lifetime sexual partners: “During your life, with how many people have you had sexual intercourse” (wave 2007). Categorical response options ranged from 1 (1 person) to 8 (21 or more people). Those who reported never had sexual intercourse were considered as having 0 sexual partners. The responses were used as a continuous score.

*Early sexual initiation*. A question was used to assess the age of sexual initiation: “How old were you when you had sexual intercourse for the first time” (wave 2007). Categorical response options ranged from 1 (13 years or younger) to 9 (21 years or older). Those who had first sex at 15 years of age or younger were considered as having early sexual initiation [12].

*Sexually transmitted infections (STIs).* Participants reported whether they had been told by a health care provider that they had chlamydia, HPV, genital warts or any STIs (wave 2013). Those who responded affirmatively were considered as having STIs diagnosis.

*Short sleep duration.* A question was used to assess sleep duration: “In a typical 24-hour period, how many hours of sleep do you get?” Response options ranged from 1 (less than 5 hours) to 8 (11 or more hours). Short sleep duration was defined as <7 hours of sleep over a 24-hour period [13].

*Preventive healthcare use.* Participants’ use of preventive healthcare was assessed with the question: “When was your last routine (preventive) physical exam or check-up?” Response categories ranged from 1 (past year) to 3 (more than 2 years ago). Responses were dichotomized as past-year use (1: past year) or no past-year use (2: past 1 to 2 years, 3: more than 2 years ago).

Physical Health

*Overweight or obese*. Participants reported their height (in inches) and weight (in pounds) (wave 2010), based on which their body mass index (BMI, kg/m^2^) was calculated. BMI≥25 kg/m^2^ was defined as overweight or obese [14].

*Number of physical health problems.* Participants reported whether they had ever been told by a health care provider that they had cancer, diabetes, high cholesterol (or high triglycerides or lipids), high blood pressure or asthma (wave 2010). A summary score was created as the total number of physical health problems reported.

**REFERENCES**

1. Conceptualization and measurement of health for adults in the health insurance study: Rand Corporation; 1979.

2. Rosenberg M. Society and the Adolescent Self-Image. Princeton, NJ: Princeton University Press; 1965.

3. Sinclair SJ, Blais MA, Gansler DA, Sandberg E, Bistis K, LoCicero A. Psychometric Properties of the Rosenberg Self-Esteem Scale: Overall and Across Demographic Groups Living Within the United States. Evaluation & the Health Professions. 2010;33(1):56-80. doi: 10.1177/0163278709356187.

4. Stanton AL, Kirk SB, Cameron CL, Danoff-Burg S. Coping through emotional approach: scale construction and validation. J Pers Soc Psychol. 2000;78(6):1150-69. PubMed PMID: 10870915.

5. Andresen EM, Malmgren JA, Carter WB, Patrick DL. Screening for depression in well older adults: evaluation of a short form of the CES-D (Center for Epidemiologic Studies Depression Scale). Am J Prev Med. 1994;10(2):77-84. PubMed PMID: 8037935.

6. Muris P, Merckelbach H, Ollendick T, King N, Bogie N. Three traditional and three new childhood anxiety questionnaires: their reliability and validity in a normal adolescent sample. Behav Res Ther. 2002;40(7):753-72. PubMed PMID: 12074371.

7. Seligman LD, Ollendick TH, Langley AK, Baldacci HB. The utility of measures of child and adolescent anxiety: a meta-analytic review of the Revised Children's Manifest Anxiety Scale, the State-Trait Anxiety Inventory for Children, and the Child Behavior Checklist. J Clin Child Adolesc Psychol. 2004;33(3):557-65. doi: 10.1207/s15374424jccp3303_13. PubMed PMID: 15271613.

8. Reynolds CR, Paget KD. Factor analysis of the revised Children's Manifest Anxiety Scale for blacks, whites, males, and females with a national normative sample. J Consult Clin Psychol. 1981;49(3):352-9. PubMed PMID: 7276324.

9. Breslau N, Peterson EL, Kessler RC, Schultz LR. Short screening scale for DSM-IV posttraumatic stress disorder. Am J Psychiatry. 1999;156(6):908-11. doi: 10.1176/ajp.156.6.908. PubMed PMID: 10360131.

10. Roberts AL, Rosario M, Corliss HL, Koenen KC, Austin SB. Childhood gender nonconformity: a risk indicator for childhood abuse and posttraumatic stress in youth. Pediatrics. 2012;129(3):410-7. Epub 2012/02/22. doi: 10.1542/peds.2011-1804. PubMed PMID: 22351893; PubMed Central PMCID: PMC3289524.

11. Field AE, Sonneville KR, Crosby RD, et al. Prospective associations of concerns about physique and the development of obesity, binge drinking, and drug use among adolescent boys and young adult men. JAMA Pediatrics. 2014;168(1):34-9. doi: 10.1001/jamapediatrics.2013.2915.

12. Dodson NA, Corliss HL, Sarda V, Emans SJ, Field AE. Sense of Mission and Sexual Health Outcomes Among Young Adult Women. Journal of Pediatric and Adolescent Gynecology. 29(6):567-70. doi: 10.1016/j.jpag.2015.03.005.

13. Steptoe A, Peacey V, Wardle J. Sleep duration and health in young adults. Arch Intern Med. 2006;166(16):1689-92. Epub 2006/09/20. doi: 10.1001/archinte.166.16.1689. PubMed PMID: 16983045.

14. WHO. Physical status: the use and interpretation of anthropometry. Report of a WHO Expert Committee. Technical Report. 1995 0512-3054 (Print) 0512-3054 (Linking).
